# The construction of a measure of behavioural complexity as a potential individual-based welfare indicator and its theoretical validation

**DOI:** 10.1017/awf.2024.48

**Published:** 2024-11-11

**Authors:** Christina Raudies, Lorenz Gygax

**Affiliations:** Humboldt-Universität zu Berlin, Department of Life Sciences, Albrecht Daniel Thaer Institute of Agricultural and Horticultural Sciences, Animal Husbandry and Ethology, Unter den Linden 6, 10099 Berlin, Germany

**Keywords:** aggregated complexity measure, animal-based welfare indicator, animal welfare, behavioural sequences, microsimulation, Shannon diversity index

## Abstract

Behavioural complexity is likely to reflect how animals cope with their environment. A large behavioural repertoire and the ability to flexibly apply these behaviours provide an individual with a greater likelihood of adapting to its (captive) environment. Here, we developed a procedure to aggregate different behavioural measures into a condensed measure of behavioural complexity based on 14 features, which were previously proposed (e.g. number of behaviours, Shannon diversity index) as well as some new features of behavioural complexity (e.g. variances of within and between transition durations). To test the measure, artificial behavioural sequences with potentially varying complexity were created using an individual-based modelling approach. With a Principal Component Analysis, the features extracted from these sequences could be reduced to two components (‘general complexity’ and ‘state variability’) explaining 59.64 and 27.68% of the total variance, respectively. The effect of the aspects of the artificial behavioural sequences on ‘general complexity’ and ‘transitions variability’ were analysed using linear mixed-effects models. The number of behavioural categories and the proportion of short behavioural states had the largest effect on the components. Both components were increasing with a greater number of behavioural categories, whereas the proportion of short behavioural states the opposite effect on the components. We also tested the approach on real data-sets. The principle components were not identical to the ones from the simulation. Yet, we found consistencies and similarities in the loadings. The approach for an aggregated measure of behavioural complexity developed here could form the basis of an individual-based animal welfare indicator for intensively kept animals.

## Introduction

Current approaches in animal-based welfare assessment, such as used in the Welfare Quality® Protocol® (Welfare Quality® 2009) or the ’Tierschutzindikatoren,’ consider specific individual aspects, such as behaviour (Schrader *et al.* 2020). Although examining individual behaviours is a valid approach, as they can indicate chronic or acute stress (Dybkjær 1992; Young *et al.* 2012) and provide insights into illness and pain (Hart 1987; Tizard 2008; Gleerup *et al.* 2015), it is essential to recognise that good health serves only as the foundational requirement for animal welfare. Ultimately, animal welfare is contingent on animals being able to fulfil their needs and satisfy underlying motivations adequately (Gygax 2017; Gygax & Hillmann 2018). Accordingly, a measure for a broader set of behaviours and how they are combined by an animal could prove useful.

A common and general problem with these welfare assessment protocols lies in the question of how the different indicators that measure widely varying aspects are combined, e.g. the Welfare Quality® score and the QBA correlated only poorly in cattle (Andreasen *et al.* 2014). Often, some weighing of different aspects by experts is used (Czycholl *et al.* 2015), which may include subjective aspects and thereby lead to faulty welfare scores (Browning 2022). Thus, an indicator that may be more complex to assess but self-evidently assimilates into one score could provide a welcome alternative to measuring many simple indicators that are difficult to be merged into one score. Here, we propose that assessing behavioural sequences in respect to their complexity may be such an approach.

Understanding and measuring complexity is a concept that has been of great interest in science (Mazzocchi 2008; Mesjasz 2010; Sherrington 2010). For ethologists, behavioural complexity is of special interest since it may allow us to capture how animals cope with their natural environment and also may provide an insight into an animal’s welfare in a captive setting (artifical environment; Cole 1995; Alados & Huffman 2000; Macintosh *et al.* 2011). A large behavioural repertoire and the ability to flexibly apply these behaviours increases the capability of an individual to adapt to its environment be it in the wild or in captivity. Behavioural complexity as an animal welfare indicator may also extend beyond currently used methods as it not only reflects its health status (Macintosh *et al.* 2011) but also its capability to cope with its environment (Asher *et al.* 2009). Another advantage of behavioural complexity as an animal welfare indicator is that a reduction in complexity may occur much earlier than would otherwise be evident from a change in the duration or frequency of a behaviour as reflected in conventional indicator variables (Rutherford *et al.* 2006; Asher *et al.* 2009). With these aspects in mind, a compact measure of behavioural complexity could have potential as an individual-based animal welfare indicator.

But what have previous views on complexity consisted of and how can complexity be measured? Here, we follow the ‘organisational’ complexity view proposed by Sambrook and Whiten (1997) due to it being more biologically relevant than the classical algorithmic complexity approach of Chaitin (1975). The latter is focused on the information required to describe a process. This information is largest in a random process because the process cannot be abstracted at all. Viewing a random process as maximally complex does not seem meaningful for a biological process. As such and taking an organisational view, complexity follows a bell-shaped trajectory increasing from simplicity (pure determinism) to a maximum and then decreasing towards full randomness (Chaitin 1975; Sambrook & Whiten 1997). The number of events in a sequence and their dependencies are crucial determinants of where on this simplicity-randomness continuum a sequence will be found (Shannon 1948). If there are only few behaviours with a strong dependency in a sequence, it is possible to formulate specific and near-deterministic rules of how the behavioural events will develop over time and this would be found on the far left of the continuum (simplicity). If there were a near-infinite number of behaviours with hardly any dependencies, sequences would be considered random, with no chance of predicting future behaviours. This sequence would be found to the far right of the continuum (randomness). For real life behaviour, the complexity often lies between these extreme poles on the simplicity-randomness axis (Reznikova 2023). Behaviour is hard to predict as it depends upon numerous internal and external factors (e.g. motivation, health, environmental conditions such as temperature; Mench 1998; Gygax 2017). However, there are certain dependencies between behaviours as they are affected by each other and one behaviour can lead to the emergence of several others with different probabilities. Behavioural sequences can therefore be considered as potentially complex systems.

There are various approaches that have been undertaken to measure the complexity of (sequential) behavioural data, such as the Shannon diversity index (Brereton & Fernandez 2022; Miller *et al.* 2020; Hall *et al.* 2021), fractal analysis (Rutherford *et al.* 2004; Maria et al. 2004; Miller *et al.* 2020), Markov-chain models (Chatfield & Lemon 1970; Macdonald & Raubenheimer 1995; Ivanouw 2007; Abner *et al.* 2013), and survival models (Gygax *et al.* 2021). The Shannon diversity index provides a simple way of calculating complexity. It describes behavioural diversity with respect to two dimensions: the quantity of different behaviours (proportion of time spent in a certain behaviour) and the evenness (distribution) across the behaviours in a sequence. Accordingly, here the main variables are summed durations of behaviours or the frequencies of bouts of the different behaviours. However, these measures do not consider sequential dependencies of the behaviours (Miller *et al.* 2020; Hall *et al.* 2021). Additionally, as the Shannon diversity index originated from the information theory (Shannon 1948), it rather follows Chaitin’s (1975) interpretation of complexity than the more biologically relevant definition of complexity from Sambrook and Whiten (1997). This means that the Shannon diversity index increases monotonously with an increasing number of behaviours. Linked to this, an animal performing only ‘basic’ behaviours could have a lower Shannon diversity index than an individual showing additional, e.g. stereotypic behaviour. The Shannon diversity index also reaches its highest values with an equal distribution of time spent in the different behavioural categories. However, animals naturally spend more time performing certain behaviours (e.g. sleep, locomotion) than others (e.g. play, agonistic behaviours). Thus, the Shannon diversity index by itself is likely to be insufficient in evaluating an animal’s welfare on theoretical grounds (Cronin & Ross 2019). Yet, the Shannon diversity index is based on relatively simple data (overall summed durations or frequencies of different behaviours) and easily calculated. These are characteristics that are attractive for a practical application.

Fractal analysis (e.g. spectral analysis, detrended fluctuation analysis) adds another dimension to the analysis of behavioural data. It can detect changes in the autocorrelation of a behaviour, i.e. the performance of a behaviour depends on the previous performance of that behaviour. The complexity of a behaviour is considered greater the higher the long-range autocorrelation (Alados & Weber 1999). The positive aspect of this method is to acknowledge dependencies of behaviour. The pitfall, on the other hand, is that different behaviours of a behavioural sequence have to be analysed separately with a binary coding (behaviour is displayed = 1, behaviour is not displayed = 0) resulting in binary sequences. It is not possible to directly generate an overall measure of behavioural complexity for different behaviours with fractal analysis, and their durations are not considered. Moreover, the analysis is relatively abstract, complicated and resource intensive.

Another approach for considering sequences of states such as behaviours are Markov-chain models. Markov-chains are stochastic models that estimate the transition probability from one state to another based purely on the previous state (Rugg & Buech 1990). Discrete-time or continuous-time Markov-chains estimate the transition probability based on the previous state as well as the time spent in that state (Rugg & Buech 1990; Anderson 1991). But as noted previously, behavioural sequences are complex systems with potential long-range dependencies. There are also Markov-chains of higher order that take longer range dependencies into account, however it can be difficult to determine the appropriate order of a model (Katz 1981). In addition, the higher the order of a Markov-chain, the higher the model’s complexity and the greater the amount of data needed to calculate the model reliably (Singer *et al.* 2014). As a result, one has to balance whether the gained accuracy of the model by higher orders compensates the added complexity of the model (Singer *et al.* 2014).

A third method that factors in the dependencies between behaviours and the durations of behaviours in a simple and straightforward way is survival analysis. As such, it is able to reflect patterns similar to a Markov-chain model or fractal analysis. It basically determines the ‘risk’ or likelihood of an individual’s transition from one behaviour to another depending on the time they have already spent in the current state. The strength of this type of analysis is the simple estimation of the model parameters and the flexibility of their application. There are no strict data requirements for the use of survival analysis. Data can be censored or uncensored and follow different distributions, e.g. exponential, log-normal, Weibull-distribution or Gamma distribution (Clark *et al.* 2003). The downside of the application’s flexibility is similar to that of the complexity of the Markov-chains of higher order; the amount of data needed to estimate these models can often not be realised with real behavioural data (Gygax *et al.* 2021).

The aim of this study was to devise an approach for creation of a new aggregated measure of behavioural complexity based on previous specific approaches. Combining different approaches with their individual weaknesses and strengths might result in an aggregated measure that has the potential to overcome the limitations of the specific approaches. We therefore included the simple Shannon diversity index (durations and frequencies of behaviours and transitions) as well as a procedure that includes sequential information (survival analysis) in our approach. We complemented these procedures with additional measures that may reflect complexity (variances in transition probabilities, median and distribution of durations and frequencies of behaviours and transitions). To test whether these single ‘features’ of complexity can be meaningfully aggregated, a Principle Component Analysis (PCA) was conducted.

For a theoretical validation of this approach, we first constructed artificial behavioural sequences, varying aspects that may have an effect on complexity. For processes that involve some aspects of unexplained variability (randomness), simulations are a useful tool. The approach of individual-based models has become widely used in behavioural biology as the underlying behavioural rules are completely known and emergent patterns can be investigated (Hemelrijk & Gygax 2004; Asher *et al.* 2009). The information obtained from the simulation can then be used to judge methods that are to be employed for analysing real world data (DeVries 2009). Here, we used a tool developed for continuous time microsimulation to create behavioural sequences (Zinn 2013). In our simulation, we varied four aspects to construct behavioural sequences: the number of behaviours; the proportion of short transitions; and the difference in duration between and within short and long transitions. The simulation provided behavioural sequences upon which we could apply our approach to measure behavioural complexity. We further assess how differences in the manipulated behavioural aspects influence our aggregated measure of behavioural complexity in terms of a sensitivity analysis. To illustrate the application of our measure of complexity, we tested our approach with three real data-sets. These data-sets already existed (Puls *et al.* 2024) and could be subjected to our approach but they were not created specifically for the purpose of this work.

In our approach, several features of complexity are to be aggregated and we used a PCA to do so. If one or more features load strongly and consistently on the measure of complexity across the simulation and the three data-sets, it is worthwhile testing whether these single features could represent the entire measure in the sense of an iceberg indicator. Such single features may be more easily applied in practice as an individual-based animal welfare indicator.

Finally, and based upon the analyses of how our measure of complexity relies on the varied aspects in the simulation, we discuss how plausibly it reflects behavioural complexity. Given the first application of our method on real-world data-sets, we can assess the feasibility of using our method in other studies. We expected our aggregated measure to mirror the potential differences in the aspects of the behavioural sequences.

## Materials and methods

### Microsimulation

To construct artificial behavioural sequences, continuous time microsimulations using the MicSim package (Zinn 2013) were run in R version 4.3.1 (R Core Team 2022). Originally, the MicSim package was designed for demographic microsimulation for sequential lifetime events (marriage, giving birth, divorce, death, etc) on a continuous time-scale. We did not use the facility of this package to define events based, e.g. on the specific age of an agent. Yet, we took advantage of that package in that events of interest, i.e. changes between states, can be modelled based on the duration of time already spent in the current state. Accordingly, we considered the lifetime events of an individual in the MicSim microsimulation as changes of behaviours in a behavioural sequence and each ‘individual’ in the model thus provided a sequence of behavioural transitions. The number of individuals per simulation run was set to 10 (see also below). The minimal model constants required by the software were the number of individuals, their sex and mortality rate. A start and a stop date also required to be set within the simulation given as the time horizon and the individuals are assigned a date of birth and an initial behaviour with which they enter the simulation and a maximum age they can reach. To model our behavioural sequences, a 50:50 sex ratio was chosen and females and males were defined identically such that there were no systematic differences between the sexes in the microsimulation (and, thus, in the behavioural sequences). The mortality rate was set to 0, i.e. no premature deaths within the simulation were possible, correspondingly no interruptions of behavioural sequences could occur for our application. The maximum age was set to a high value of 100 as no individuals were supposed to ‘die’ during the simulation. The date of birth of the individuals was set to 01.01.2010, a date chosen arbitrarily under the premise that it was at least one year but less than 100 years before the starting date.

The smallest time unit in the MicSim simulation is one day (24 h). One day in the MicSim simulation was translated into 10 s in our modelled behavioural sequences. We wanted our behavioural sequences to have a duration of 24 h, which then resulted in 8,640 days in the simulation (number of 10 s intervals in 24 h). To accommodate this, the time horizon was set from 01.01.2021 to 28.04.2044. We aimed for approximately 1,000 changes of behaviours during the selected time horizon. This number was chosen based on pilot observations of five 48-h behavioural observation videos of real finishing pigs, to which we plan to apply the measure of behavioural complexity in the future. These videos were continuously analysed with the BORIS software version 7.12.2 (Friard & Gamba 2016). The number of changes of behavioural categories was counted for each 24-h period and ranged between 850 and 1,400. A general transition rate can be added into the MicSim simulation which corresponds to the time spent in a behaviour prior to changing to another. A high transition rate equals a high transition probability and therefore leads to a shorter duration of that behaviour. In the simulation, an overall transition rate of 6 resulted in approximately 1,000 state changes for each behavioural sequence. Apart from these general and constant parameters, there were additional aspects that we varied in our simulation to create behavioural sequences potentially varying in complexity.

**Figure 1.**
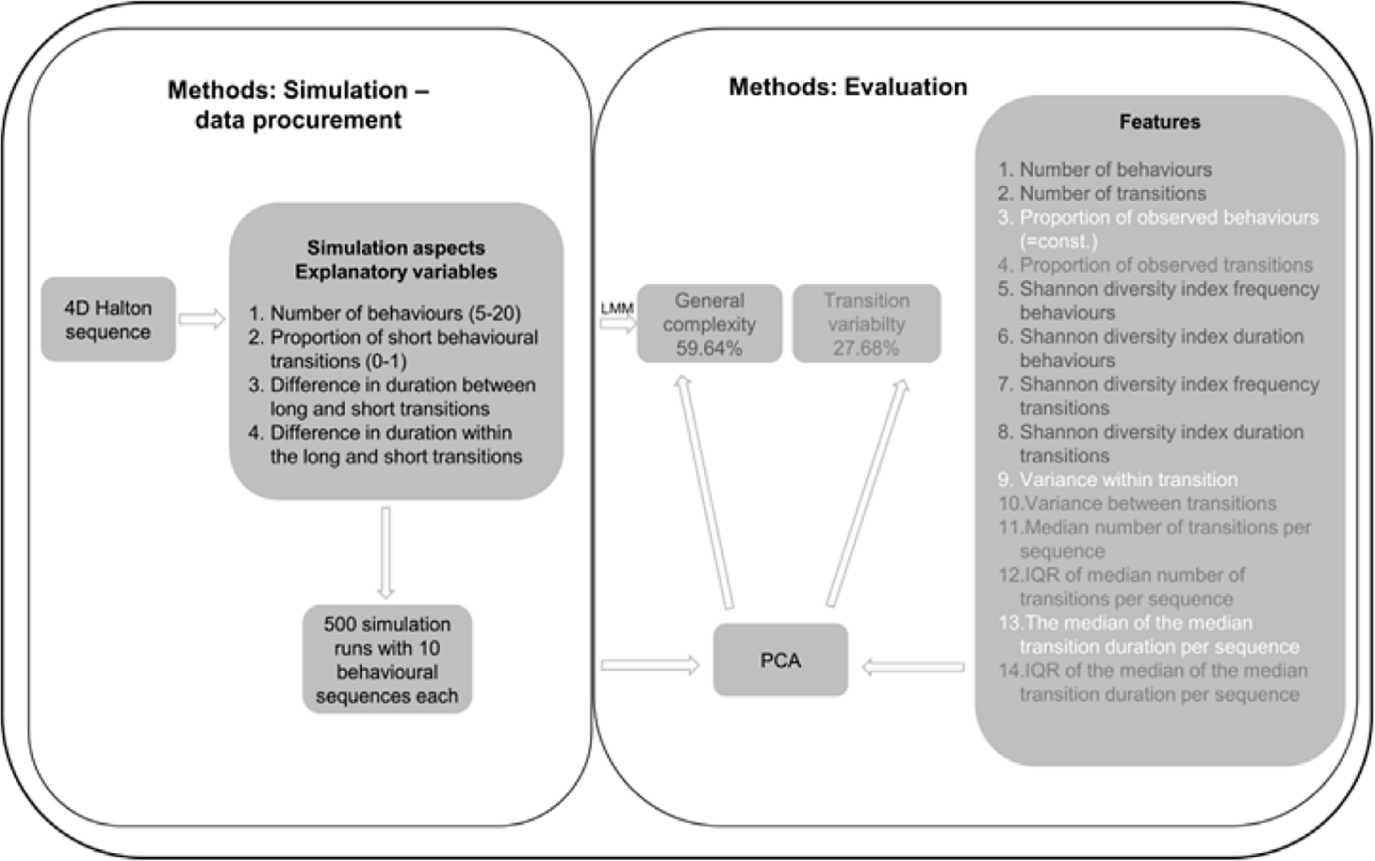
Data procurement and evaluation scheme. On the left are the important steps of the simulation procedure. The four aspects of generating the artificial behavioural sequences were varied using a four-dimensional Halton Sequence. To the right the procedure of the development of the measure of complexity is schematically depicted. The measure of complexity contained 14 different features, which were reduced into two principal components. The effects of the explanatory variables (simulation aspects) on the outcome variables (principal component 1 and 2) were analysed using mixed effect models. The grey shade of the features corresponds to the principal components based on high values of the loadings. The white-coloured features did not load on any of the two first components (according to our cut-off) or were constants and therefore not included in the PCA (also see Table 2 and *Microsimulation* and *Measure of complexity* for detailed information).

The left side of Figure 1 provides a schematic overview of the data procurement procedure and this will be described in more detail as follows. In the following, the term ‘transition’ is used to describe a specific transition between two behaviours (a single cell in Table 1). The aspects of the simulation that we varied were the number of behaviours, the proportion of ‘short’ transitions (= high transition probability, fast transition, short duration), the average difference of the ‘short’ and ‘long’ transitions as well as the variability of the duration within the short and long transitions. The specific values for these aspects of the behavioural sequences were varied between each simulation run and generated using a 4-dimensional Halton sequence using the R package randtoolbox (Dutang & Savicky 2023). A Halton sequence works as a random number generator and the random numbers on the (four) dimensions vary independently from each other but fill the multi-dimensional space spanned by the variables evenly. All dimensions in the Halton sequence could take continuous values between 0 and 1 (Young 2016).

**Table 1.** Transition matrix between current behaviours a–n (column) and following behaviours a–n (row) providing definitions of the variables used in the definition of the features of behavioural complexity (see text). Behaviours were chosen to be mutually exclusive and could only stop when another behaviour occurred, which leads to an empty diagonal. d indicates the duration of the corresponding transition represented by that particular cell

Following	a	b	c	…	n
Current					
a		d_ab_ short	d_ac_ short	…	d_an_ long
b	d_ba_ short		d_bc_ long	…	d_bn_ long
c	d_ca_ short	d_cb_ short		…	d_cn_ short
…	…	…	…		…
n	d_na_ long	d_nb_ short	d_nc_ short	…	

The first dimension of the Halton sequence was used for the number of behaviours (a to n in Table 1), which we varied between 5 and 20. This range was chosen based on our ethogram used for the observation of the behavioural video analysis of fattening pigs (see above). The ethogram consisted of ten behaviours and the simulation was thus centred on this number (from half to twice this number). The numbers of the Halton-sequence (0–1) were linearly scaled to the range between 5 and 20 and rounded to a full number.

The second dimension was used for the proportion of short transitions. The values of the Halton-sequence were multiplied by the number of transitions (= n^2^-n; no transitions between the same behaviours were considered in the simulation) and rounded to a full number. The remaining transitions were assigned as long transitions (labels ‘short’ and ‘long’ in Table 1).

The third dimension in the Halton sequence was used for the variation between the short and long transitions, i.e. the difference between the average duration of the long and short transitions. We aimed at scaling the shortest durations to 10 s (as the smallest time unit in MicSim is 24 h which equalled 10 s in our simulation) and the longest durations to 2 h (reasonably long duration for behaviours that would be performed for longer periods of time, e.g. resting or exploration). These values corresponded in the simulation to rates of approximately 11 and 0.5, respectively. Accordingly, these were the extreme cases (with the overall transition rate, OTR, of 6 mentioned above in the ‘middle’) that we wanted to see in the simulation. We did not find a simple single function that translated our 0–1 values from the Halton sequence to this span of values and, accordingly, used an *ad hoc* pragmatic solution. We introduced a scaling factor of 3 (SF) and linearly scaled our Halton value (HV) to the range between 1/SF = 1/3 and 5. The average rate for the short transitions was calculated as *OTR + HV.* For the short transitions, we thus reached a range for the rate between 6 + 1/3 = 6.33 and 6 + 5 = 11. The average rate for the long transitions was calculated as *OTR/(HV × SF).* For the long transitions, we reached a range between 6/(1/3 × 3) = 6 and 6/(5 × 3) = 0.4.

The fourth dimension in the Halton sequence was used for the relative variation among the short and long transitions (within the transition labelled short and long in Table 1) and ranged between 0.05 and 0.5 times the mean duration for short and long transitions. For this, the value in the Halton sequence was linearly scaled to the range of 0.05 to 0.5.

The actual durations of each instance of a transition were varied inherently in the MicSim microsimulation on a bout-per-bout basis and was therefore not additionally and explicitly varied.

To reduce the dependency between simulated behavioural sequences, we chose a small number of individuals (behavioural sequences) in each run (see above). As such, we ran the simulation 500 times, resulting in 5,000 random artificial behavioural sequences with each simulation providing 10 sequences. The behavioural sequences were subsequently evaluated in regard to how the aspects that we varied influenced the degree of complexity, using a newly developed measure of behavioural complexity (see the following sub-section).

### Measure of complexity

The basis of the measure of complexity were 14 features that were reduced to fewer variables based on a Principal Component Analysis (PCA; Figure 1; right). The PCA was based upon the correlation matrix of the features. The most basic features were the number of observed behaviours and transitions and the proportion of observed behaviours and transitions given the maximum number of behaviours in the ethogram and the corresponding number of possible transitions.

Further features were four different variants of the Shannon diversity index (H). The index was calculated either based on the total frequency or the total (summed) duration of the behaviours or the transitions. In the following formula *p* was substituted with the according value: 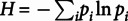 .

Two additional features of the measure of complexity were the variability of the durations within and between transitions. The ‘within’ variance refers to the variance of the duration of repeated transitions (the within cell variance in Table 1 averaged across all transitions). The ‘between’ variance refers to the variance of the duration between transitions (the between cell variance in Table 1). These two features were calculated based on a mixed-effects model with the logarithm of the durations of the single bouts as the outcome variable, an intercept as the fixed and the transitions as the random effect. The between transition variance was calculated by the variance component of transition and the within transitions’ variance by the model’s error. R version 4.3.1 (R Core Team 2022) (R Core Team 2022)and the blme package (Chung *et al.* 2013) based on lme4 (Bates *et al.* 2015b) was used for this.

Finally, the median of the number of bouts across the different transitions and its interquartile range, and the median of the median duration of bouts across transitions and its interquartile range were features included in the measure of complexity.

### Real data-sets

To apply our approach to real data-sets, we used the data collected in three small studies, in which animals could visit eight cages with different resources from a central choice cage. Here, we only present the relevant aspects of these studies, see Puls *et al.* (2024) for the detailed description of the methods. In these studies, single rats (n = 11), rat groups of three animals (four groups) and hen groups of three birds (four groups) were observed. In all experiments, feed, water, resting, and foraging opportunities as well as novel objects and olfactory cues of a predator were available in one resource cage each. Rats were provided with a running wheel and single rats afforded the opportunity for restricted contact with social partners, with groups of rats given an empty control cage. Additionally, the hens were provided with a nest to lay eggs and an area to sand-bath.

The animals spent ten consecutive days in the experimental system. Data were recorded automatically each day for the single rats and data based on video recordings from days 1, 3, and 6 for the rat and hen groups. We considered the duration from the entry to one resource to the entry of the following resource cage (possibly the same) as the behaviours on which we based the features for our complexity measure in the same way as for our simulated data. In these data-sets, behaviours were not observed in detail but each cage provided a specific behavioural context and was thought to serve a specific motivation. This means that, for these illustrative examples, we did not calculate the complexity of behaviour in a narrow sense but focused on the complexity of changes between different motivations. We conducted the PCA individually for the three real data-sets in order to ascertain whether the loadings of the principal components resemble each other in different data-sets.

These studies were approved by the university’s animal welfare officers and by the responsible veterinary office (LAGeSo Landesamt für Gesundheit und Soziales, Berlin; rats, individual testing: permit no. G003/20; rats, small groups: StN 002/21; hens, small groups: StN 0012/21).

### Statistical analysis

The statistical analysis was conducted with R version 4.3.1 (R Core Team 2022). To reduce the number of features of the measure of complexity, a PCA was applied (Abdi & Williams 2010) for the simulated data as well as the real data-sets based on the correlation structure. To achieve a univariate normal distribution for the features as closely as possible prior to including them into the PCA, they were transformed (Table 2). For any PCA, a decision needs to be taken on how many variables to further consider. The number of principal components (PC) that we chose here (two for the simulated data and three for the real data-sets) represented a reasonable balance between the cumulative variance explained on the one hand, and a small number of PCs with an ease of interpretation on the other. This interpretation was specifically simple if we used an (arbitrary) threshold of 0.3 for the loadings (Table 2). Moreover, the PCs considered had all an eigenvalue greater than 1 (and all other PCs smaller than 1).

**Table 2.** Principal Component analysis on the features of behavioural complexity: proportion of variance, cumulative variances, and Eigenvalues of as well as factor loadings on the first two and three principal components for the simulation and the three pilot data-sets, respectively. Numbers in bold indicate loadings for which the absolute value was above a cut-off value of 0.3. We focused on interpreting loadings with values ≥ 0.3, however the remaining loadings were not neglected in the process of component calculation

Data-set Component No.	Transfor-mation	Simulation		Rats, individuals			Rats, groups			Hens, groups		
		1^A^	2^B^	1	2	3	1	2	3	1	2	3
Proportion of variance [%]		59.64	27.68	39.74	18.92	13.39	46.86	15.13	12.78	45.54	20.54	14.60
Cumulative variance [%]		59.64	87.32	39.74	58.67	72.06	46.86	62.00	74.76	45.54	66.09	80.69
Eigenvalues		7.75	3.60	4.37	2.08	1.47	4.37	1.66	1.41	5.47	2.46	1.75
Number of observed behaviours	log	**0.323**	0.221	Const.^1^	Const.	Const.	Const.	Const.	Const.	Const.	Const.	Const.
Number of observed transitions	log	**0.345**	0.137	(1)^2^	(1)	(1)	(1)	(1)	(1)	(1)	(1)	(1)
Proportion of observed behaviours	arcsin–sqrt	Const.	Const.	Const.	Const.	Const.	Const.	Const.	Const.	Const.	Const.	Const.
Proportion of observed transitions	arcsin–sqrt	0.020	**–0.417**	**0.416**	0.160	0.177	**0.407**	0.044	0.092	**0.327**	0.131	0.155
Shannon diversity index (SDI) frequency behaviours	none	**0.351**	0.072	**0.356**	**0.305**	0.221	**0.409**	0.006	0.097	**0.374**	–0.035	0.271
SDI duration behaviours	none	**0.352**	0.045	0.233	**–0.347**	**0.482**	**0.305**	–0.274	–0.139	0.249	**–0.409**	–0.290
SDI frequency transitions	none	**0.334**	0.186	0.133	**0.474**	0.280	0.286	–0.136	**0.356**	**0.311**	–0.043	**0.457**
SDI duration transitions	none	**0.343**	0.118	0.145	**–0.478**	**0.414**	0.105	**–0.708**	–0.067	0.266	**–0.512**	–0.053
Variance within a transition	log	–0.239	0.194	0.164	–0.291	**–0.337**	–0.105	0.079	**–0.625**	–0.152	**0.352**	**0.439**
Variance between transitions	log	–0.242	**0.319**	–0.156	**0.437**	0.141	–0.220	**0.307**	**0.516**	–0.114	–0.268	**0.376**
Median number of transitions	none	–0.027	**–0.477**	**0.388**	0.072	0.061	**0.383**	–0.028	0.178	**0.314**	0.078	0.193
IQR^3^ of the median number of transitions	none	–0.079	**–0.386**	**0.366**	–0.116	–0.081	0.240	0.172	**–0.339**	**0.367**	–0.060	–0.174
Median of the median transition duration	log	–0.293	0.250	**–0.387**	–0.112	**0.332**	–0.294	**–0.464**	0.160	–0.247	**–0.448**	0.146
IQR of the median of the median transition	log	–0.252	**0.352**	**–0.350**	0.001	**0.428**	**–0.362**	–0.237	0.057	–0.205	**–0.369**	**0.424**

A General Complexity

B Transition variability

1 “Const.” indicates features that were (nearly) constant and showed no variability, i.e. all potential behavioural categories or transitions did occur.

2 (1) The maximum number of possible transitions was constant; therefore, the number of transitions and the proportions of transitions are equivalent

3 IQR=interquartile range.

For the simulated data, the effects of the explanatory variables (aspects of the simulation: number of behaviours, proportion of short transitions, difference of the duration between and within short and long transitions) and their interactions on the outcome variables (principal component 1 and 2) were analysed using linear mixed-effects models based on the function lme (packages nlme; Bates *et al.* 2015a). To do so, these continuous explanatory variables were normalised. The random effect was the simulation run. We calculated model estimates based on the package contrast (O’Callaghan *et al.* 2022) and used the ANOVA function for calculating *P*-values.

For the real-data sets, the features were calculated for each observation day and the PCAs were conducted based on the daily values (separately for each experiment). The effect of day that the animals had spent in the experimental system (factor with ten levels for the individual rat data and with three levels for the group data) on the outcome variables (principal components 1 to 3) were analysed per component and experiment using linear mixed-effects models based on the packages blme (see above). The random effect consisted of the individuals nested in group and, for the group observations, crossed with observation day. We used a parametric bootstrap for model estimates and confidence intervals (function bootMer provided in lme4) as well as for calculating *P*-values (package pbkrtest; Halekoh & Højsgaard 2014).

Model assumptions (normal distribution, homoscedasticity) were checked via graphical analysis of the residuals. No major deviations from the assumptions were observed.

As a simplification in respect to assessing the complexity of the behavioural sequences we re-ran these analyses of the simulated data and the real data-sets using the Shannon diversity-index as calculated on the frequency of the behaviours as the outcome variable. This diversity index was chosen because it loaded strongly on the first principal component in all our data-sets (see *Results*) and because it is a relatively simple measure based on counting how many bouts of each behaviour occurred (see *Discussion*). Given these characteristics, the Shannon index could serve as an ideal iceberg indicator for the overall aggregated complexity measure (principal components).

## Results

### Simulated sequences

The PCA showed that most of the variables loaded strongly on either the first or the second component (Table 2). The number of observed behaviours, the number of observed transitions and the Shannon diversity indices loaded positively on PC1. The first principal component was thus referred to as ‘general complexity’ as it contained most of the currently established aspects to describe behavioural complexity. The variance between transitions and the interquartile range of the median of the median transition duration loaded positively on PC2 whereas the proportion of observed transitions, the median number of transitions, and the interquartile range of the number of transitions loaded negatively on PC2 (Table 2). Due to the high positive loadings of the features regarding the variability of the transitions the second principal component was called ‘transition variability’.

**Figure 2.**
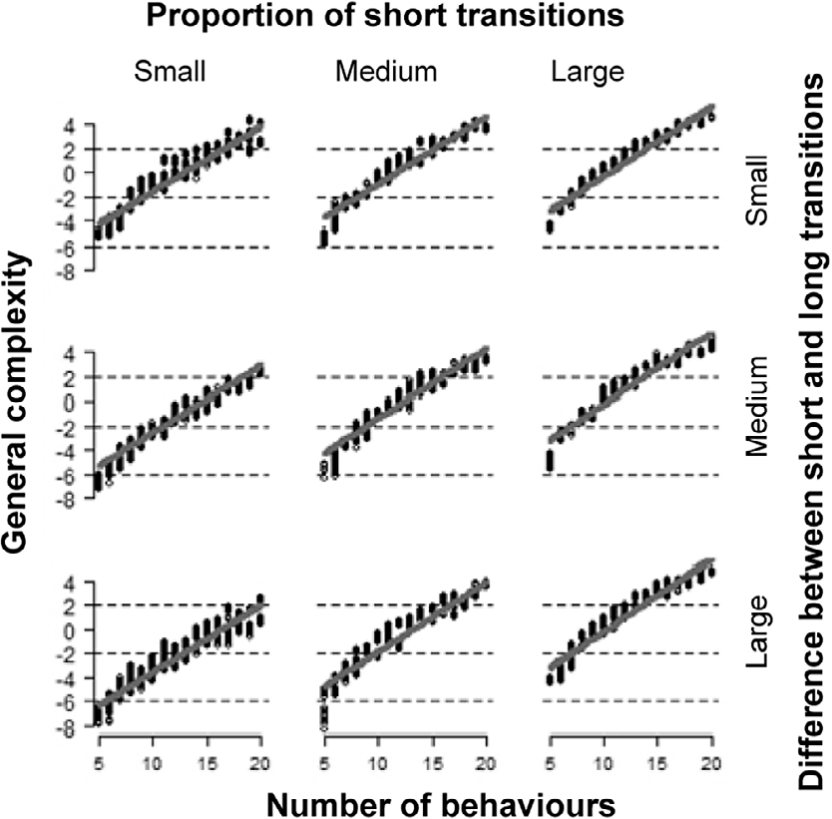
Effect of the number of behaviours, the proportion of short transitions and the difference in the duration of the transitions on the general complexity (see Table 2). The proportion of short transitions and the difference in the duration of the transitions were continuously varied in the simulation and divided each into three equal parts for illustration based on the 33rd (1/3) and 67th (2/3) percentiles. The grey line shows the model estimate and the dashed lines show the (very narrow) 95% confidence intervals. The model estimate is based upon the mid-point of the percentiles mentioned above, i.e. the 16th (1/6), 50th (3/6) and 83rd (5/6) percentiles.

The general complexity increased with an increasing number of behaviours and was higher for large proportions of short transitions and slightly higher for small differences in the duration between short and long transitions (Figure 2, Table 3). The general complexity decreased slightly with an increasing relative variance among the short and the long transitions. Yet, the strength of this relationship was negligible (Figure S1; left, Table 3).

**Figure 3.**
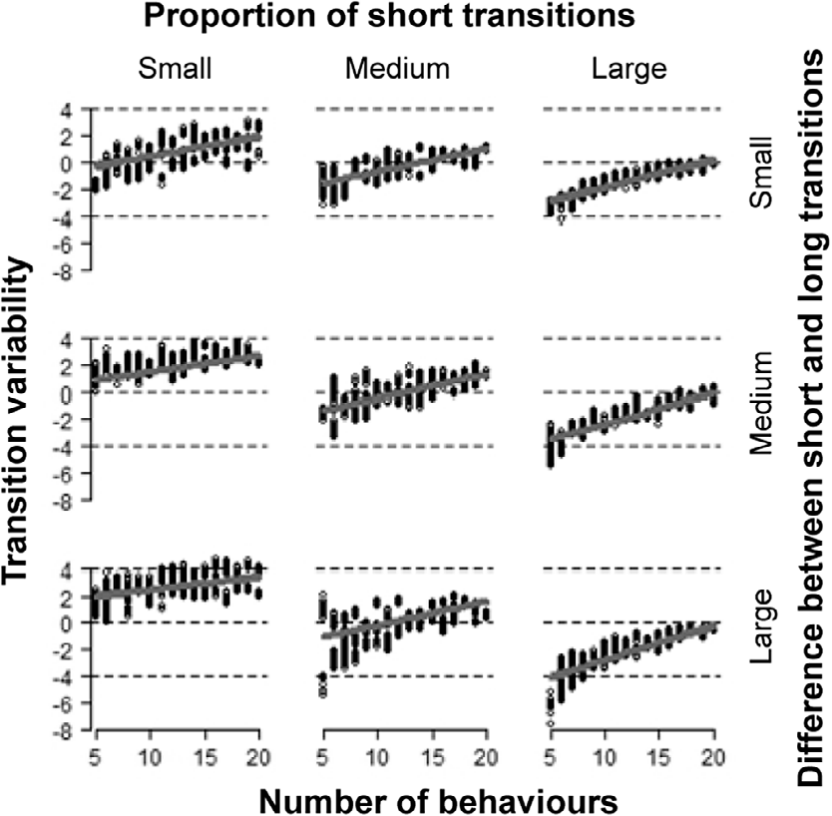
Effect of the number of behaviours, the short transitions and the difference in the duration of the transitions on the transition variability (see Table 2). The proportion of short transitions and the difference in the duration of the transitions were continuously varied in the simulation and divided each into three equal parts for illustration based on the 33rd (1/3) and 67th (2/3) percentiles. The grey line shows the model estimation and the dashed lines show the (very narrow) 95% confidence intervals. The model estimation is based on the mid-point of the percentiles mentioned above, i.e. the 16th (1/6), 50th (3/6) and 83rd (5/6) percentiles.

**Table 3.** Detailed *P*-values of the linear mixed effects models. Numbers in bold indicate *P*-values ≤ 0.05

	dfs	General complexity		Transition variability		SDI frequency behaviours	
		F	*P*	F	*P*	F	*P*
Global test^1^	15	**1591.850**	**<0.0001**	**1313.320**	**<0.0001**	**1667.87**	**<0.001**
Main effects							
Number of behaviours (numBehav)	1,484	**9409.298**	**<0.0001**	**1115.601**	**<0.0001**	**115.41**	**<0.0001**
Number of short transitions (numShort)	1,484	**1672.913**	**<0.0001**	**4556.275**	**<0.0001**	**6.74**	**<0.0001**
Difference in duration between short and long transitions (diffDur)	1,484	**218.323**	**<0.0001**	**108.630**	**<0.0001**	**–5.29**	**<0.0001**
Relative variance between short and long transitions (relVar)	1,484	**9.023**	**0.0028**	**6.802**	**0.0094**	–0.87	0.387
2–way interactions							
numBehav:numShort	1,484	**9.046**	**0.0028**	**82.275**	**<0.0001**	0.20	0.844
numBehav:diffDur	1,484	1.087	0.2978	0.176	0.6746	1.13	0.261
numBehav:relVar	1,484	0.824	0.8240	1.412	0.2353	**4.34**	**<0.0001**
numShort:diffDur	1,484	**234.946**	**<0.0001**	**473.906**	**<0.0001**	0.19	0.853
numShort:relVar	1,484	0.550	0.3644	0.024	0.8770	–0.19	0.850
diffDur:relVar	1,484	0.075	0.4548	0.284	0.5942	0.29	0.769
3–way interactions							
numBehav:numShort:diffDur	1,484	0.004	0.9503	**13.458**	**0.0003**	–1.07	0.287
numBehav:numShort:relVar	1,484	0.005	0.9427	0.165	0.6849	–0.30	0.766
numBehav:diffDur:relVar	1,484	2.997	0.0841	0.009	0.9238	–0.69	0.488
numShort:diffDur:relVar	1,484	0.903	0.3425	0.239	0.6250	1.47	0.142
4–way interaction							
numBehav:numShort:diffDur:relVar	1,484	0.506	0.4771	3.280	0.0708	–1.71	0.089

1 Likelihood-ratio test with a 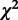 test statistic listed in the column of the F-value

The transition variability increased with the number of behavioural categories. This relationship was weaker with an increasing proportion of short transitions and a higher difference between short and long transitions. Moreover, the transition variability was higher for small proportions of short transitions. (Figure 2, Figure 3, Table 3). Finally, the transition variability increased slightly with an increasing relative variance among the short and the long transitions. Yet, the strength of this relationship was again negligible (Figure S1; right, Table 3).

### Real data-sets

The results of the PCAs based on real data-sets showed a number of consistencies in the first principal component over the three different studies (Table 2). The proportion of observed transitions, the Shannon diversity index for the frequency of the behaviours, and the median of the number of transitions loaded strongly positive on the first component for all data-sets. In addition, the Shannon diversity index for the duration of the behaviours, and for the frequency of the transitions as well as the median number of transitions, the IQR of the median number of transitions loaded also positively on the first component. The median of the median transition duration and its IQR loaded negatively on the first component although these loadings did not surpass our chosen cut-off value in all three data-sets.

The second component consisted predominantly of the Shannon diversity index for the frequencies and the corresponding index for the durations. The variance between transitions and the median of the median transition durations also loaded negatively on this component.

The third component of the pilot data-sets did not show a clearly consistent pattern in the factor loadings. It was included for presentation here to reach a cumulative variance over 70%.

The PCs from the real data-sets were not identical with the ones from the simulation. Yet, in the first PC from the real data-sets, all Shannon indices loaded positively as for the simulation (although not all with values above our cut-off) and, in the first PC from the simulation, the median of the median transition duration and its IQR also still loaded relatively high. Consequently, at least the first PCs in all our data-sets did have some similarities.

**Figure 4.**
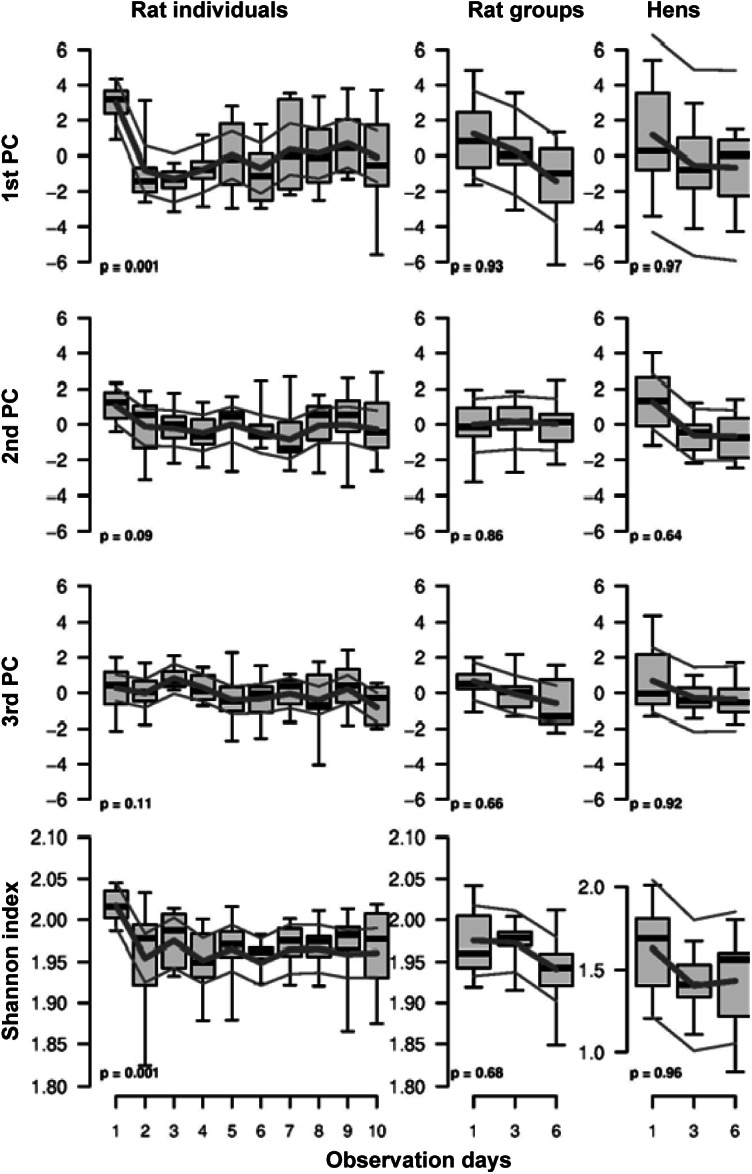
Values of the first three principal components based on the 14 features for behavioural complexity (see Table 2) and the Shannon diversity index based on the frequency of the different behaviours across days in the experiment for three real data-sets based on observations of individual rats, rats in small groups, and hens in small groups. *P*-values are given for the effects of experimental day. Boxplots show raw data (thick lines), model estimates (thin lines) 95% confidence intervals.

The values of the first PC dropped from day 1 to 2 and increased again slightly thereafter for the single rats (Figure 4), whereas the values decreased continuously for the two observations of small groups (although this decrease could not be supported statistically). The second PC showed a less clear but similar pattern. Finally, the third PC showed little consistency in the pattern over time (Figure 4).

### Shannon diversity index as a representative indicator for complexity

**Figure 5.**
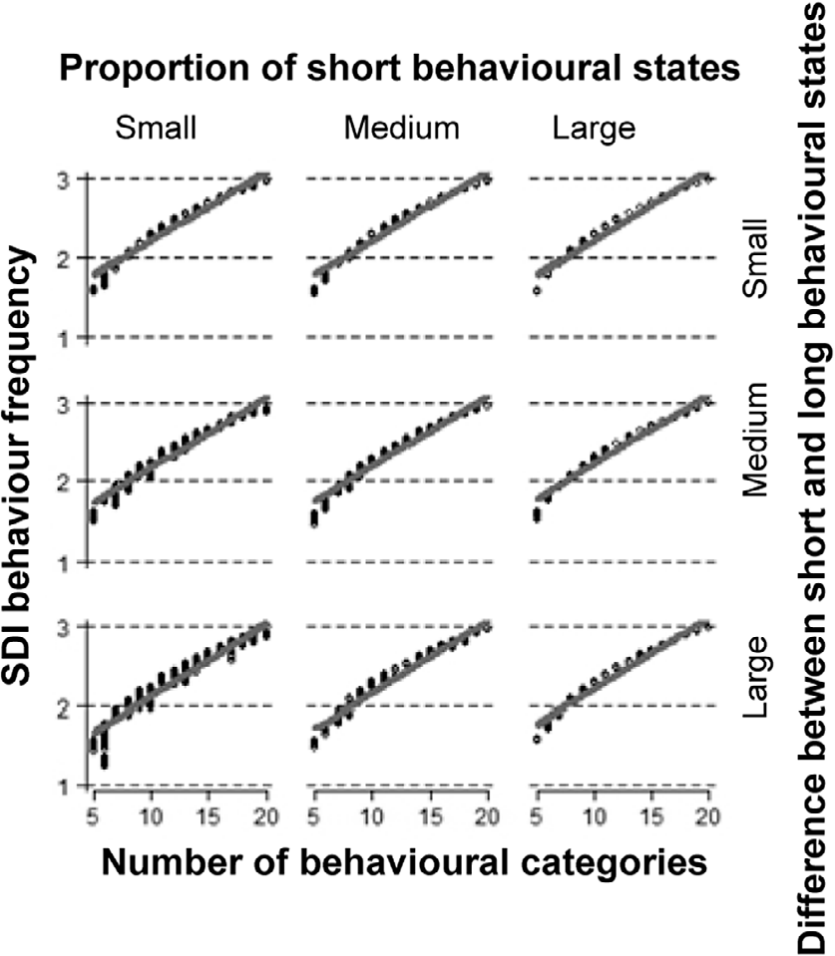
Effect of the number of behaviours, the proportion of short transitions and the difference in the duration of the transitions on the Shannon diversity index for the frequency of behaviours. The proportion of short transitions and the difference in the duration of the transitions were continuously varied in the simulation and divided each into three equal parts for illustration based on the 33rd (1/3) and 67th (2/3) percentiles. The grey line shows the model estimate and the dashed lines show the (very narrow) 95% confidence intervals. The model estimate is based on the mid-point of the percentiles mentioned above, i.e. the 16th (1/6), 50th (3/6) and 83rd (5/6) percentiles.

Due to the consistently strong positive loading of the Shannon diversity index for the frequency of behaviours on the first component throughout all data-sets (Table 2), we chose that feature as a potential representative for the ‘general complexity’, i.e. the first principal component. The SDI for the frequency of behaviours increased with an increasing number of behaviours and was higher for a larger proportion of short transitions (*F*_1,484_ = 45; *P* < 0.001) and slightly higher for small differences in the duration between short and long transitions (*F*_1,484_ = 28; *P* < 0.001) (Figure 5). In the real data-sets, the evolution of the Shannon diversity index across time was highly similar to the evolution of the first principal component (Figure 4 bottom row).

## Discussion

The aim of this work was to develop an approach to create an aggregated measure of behavioural complexity and to validate it theoretically using artificially generated behavioural sequences of varying complexity and to illustrate its use with exemplary real data-sets. In our simulations, we were able to come up with two dimensions (‘general complexity’ and ‘transition variability’) which explain 87% of the total variability contained in our 14 features of complexity. For that reason, these two components yielded a compact measure of behavioural complexity. In the sense of a sensitivity analysis, we found that changes in the aspects of the artificial behavioural sequences that were assumed to account for differences of behavioural complexity did translate into a corresponding change in our two components. The number of behaviours, the proportion of short transitions and the difference in the duration between long and short transitions had the greatest effect on both components.

Both components increased with an increasing number of behaviours. Indeed, it has been proposed for a long time, that the number of behaviours in an animal’s behavioural repertoire is an indicator for behavioural complexity (Sambrook & Whiten 1997). It has been stated more generally that complexity depends on the amount of information needed to specify the system concerned (Shannon 1948). A behavioural sequence containing only two behaviours requires less information to be described than a sequence containing, for example, six different behaviours. Our results, therefore, support the notion that the “*number of acts in an animal’s behavioural repertoire should index its behavioural complexity*” (Sambrook & Whiten 1997). This notion is heavily focused upon the potential of an animal to perform different behaviours. Yet, in real-life data, the number of behaviours might be limited by environmental factors and by the level of detail of the ethogram used. If environmental factors are similar between conditions of interest, a clear difference in the number of behaviours may not be expected. Based on a detailed ethogram it was found that sows in restricted environments only show 30% of the behaviours compared to sows in semi-natural environments (Stolba *et al.* 1983). Yet, if an ethogram is defined in less detail due, for example, to practical feasibility, the chances of detecting differences in the number of behaviours are relatively low or only possible with extreme differences of conditions. This is specifically true if the behaviours considered satisfy basic needs, which can be expected in almost all circumstances. As a result, differences may only become apparent with more nuanced ethograms including behaviours such as comfort and play behaviour or even subdividing these further into grooming and allo-grooming, for example. Then, differences in the number of behaviours performed could be expected also between housing systems that differ little as, for example, the space allowance could inhibit some behaviours such as play behaviour as shown in calves (Jensen *et al.* 1998; Jensen & Kyhn 2000). The level of detail of the ethogram used for real-life data is crucial for the possibility of detecting differences in the number of behaviours. However, the level of detail of an ethogram is fixed within a given study and only relative comparisons can and need to be made, such as relative differences in behavioural complexity between housing conditions or within the same animal.

The feasibility of the analysis of behavioural sequences based on a more detailed ethogram will decrease with higher levels of details. The future application of computer-based, video-analysing tools, such as DeepLabCut (Mathis *et al.* 2020), could help to reduce the workload related to manual video analysis and enable an increased level of detail for ethograms and, as a result, detecting complexity in behavioural sequences based on the number of behaviours. In our real data-sets with eight different behaviours, the number of behaviours were constant and no difference in the number of behaviours was detectable across time. It is advisable therefore to choose an ethogram as detailed as possible for analysing the behavioural sequence while still being feasible.

The proportion of short transitions and the difference in the duration between short and long transitions were aspects that also affected the general complexity and the transition variability although with opposite signs. The general complexity was higher with an increasing proportion of short transitions and smaller differences in the duration between short and long transitions. A high number of short transitions leads to a higher number of transitions in a 24-h period. The more transitions taking place, the greater the chances for different transitions to be observed, which contributes to the overall variability of a behavioural sequence. The general complexity contained four different Shannon diversity indices. These indices have their highest value with the greatest homogeneity among the behaviours. This is in alignment with a higher general complexity for small differences in the duration between short and long transitions. Concerning the transition variability, a smaller proportion of short transitions and larger differences in the duration between short and long transitions lead to higher values for this component. This component has higher values for behavioural sequences with less transitions, if there is greater variability in the durations within and between the transitions. This could be seen in the high loadings for the ‘variance between transitions’ and the ‘IQR of the median of the median duration of transitions’ which both positively loaded on the transition variability. The general complexity reflects the number of transitions as the main contribution, whereas the transition variability maps the variance of the transitions as the main contribution to the overall varying behavioural complexity of a behavioural sequence.

Perhaps unsurprisingly, the patterns were somewhat less clear for the real data-sets. Yet, the original variables loading relatively highly on the first principal component were the same as for the simulation even if the loadings were somewhat smaller. Moreover, the changes in behavioural complexity over time are plausible specifically for this first component as it is reasonable to assume that the behavioural complexity increased when an animal was introduced to a new environment in order to adjust to the new surroundings. A similar reaction in hens was shown previously. The behavioural complexity of vigilance behaviour increased in hens when entering a novel arena (Rutherford *et al.* 2003). For the single rats, we can rule out that the high initial values for complexity being due to a stress response since no elevated faecal stress markers were found (Puls *et al.* 2024). The behavioural complexity in our pilot data-set then decreased with habituation to the environment. A decrease in behavioural complexity due to a lack of unpredictability and novelty was described before in relation to stereotypic behaviour (Mason & Rushen 2008). In our data-sets, the behavioural complexity increased again for the individual rats towards the end of the test period. A similar effect has been shown previously due to latent learning (Blodgett 1929; Tolamn 1948). After a few days in a maze, the behaviour of rats seemed to be random (decrease in behavioural complexity due to a lack of novelty, see previously). However, in the next phase of their experiment when they introduced a reward, a change in the rats’ behaviour again became apparent, with them moving in a goal-orientated way (Christensen 2004). Learning can lead to the modification, discard or generation of new behavioural rules depending on its relevance in the current environmental conditions (Inglis & Langton 2006). The slight differences in the behavioural complexity over time, i.e. an increase of behavioural complexity after initial decrease in individual but not group-tested rats, may be due to the observation windows. The individual rats were observed for ten consecutive days whereas the groups of hens and rats were only observed on days 1, 3 and 6. The behavioural complexity of the groups may possibly have increased again had an additional observation on day 10 been included. A difference between the pattern between rats and hens could also be explained by different motivations to explore. Rats are thought to have a high intrinsic motivation for exploration (Hughes 1997) whereas chickens as typical prey animals can be more wary and less explorative when faced with new stimuli (Calder & Albright 2021)

Overall, most of our features of complexity did show a relatively strong loading on either of the two first components, indicating that the approach to aggregate the features is useful. The Shannon diversity index for the frequency of behaviours loaded consistently high on the first principal component in all the data-sets and the analysis of that feature alone did show the same pattern as the ‘general complexity’ and was mainly driven by the number of behaviours in the simulation. The Shannon diversity index for the frequency of behaviours could thereby function as a potential iceberg indicator. This measure could be observed with relatively little effort because only the number of bouts of the different behaviours needs to be recorded.

One understanding of complexity is that it is “structures that fill space and time” (Macintosh *et al.* 2011). This is a fairly abstract concept. Translated to behavioural complexity one could say that anything that alters the spatial or temporal arrangement of behavioural sequences contributes to behavioural complexity. We not only included different numbers of behavioural categories to fill the ‘space’ of our behavioural sequence but also altered different aspects of the temporal arrangement of the behaviours with the different proportions of short transitions and the variance within and between the long and short transitions. Another more explicit understanding of complexity is that complex systems have three dimensions, namely diversity, flexibility and combinability (Rebout *et al.* 2021). Diversity was described as the variation of the number of elements in a system. Flexibility is the variation within the elements of a system. Combinability is the possibility of the elements of a system interacting (Rebout *et al.* 2021). With the four aspects we chose, all three of these dimensions were considered. The diversity is given by the varying numbers of behaviours, the flexibility is given by the variation in the duration among transitions and the combinability is given by the fact that we used specific transitions as the elements of our system. Variations in these four aspects were successfully mirrored in the features of our complexity measure, which are assumed to reflect behavioural complexity. We are therefore confident that the four aspects we chose were sufficient to create behavioural sequences of varying complexity.

For our measure of complexity we only considered first-order dependencies by using transitions (= a specific transition between *two* subsequent behaviours) as the basis for most of the fourteen features contributing to our measure, although it can be assumed that not only current behaviours but also previous ones determine the following behaviour of an animal (Gygax *et al.* 2021). As mentioned in the *Introduction*, it can be difficult to determine the correct order for a model and the amount of data needed for generating reliable results with models of higher order can only rarely be realised with behavioural data (Singer *et al.* 2014). Using models of higher order would most likely have resulted in overfitting with our data. In our simulation where the number of behaviours ranged between 5 and 20, we would have ended up with 125–8,000 different possible transitions assuming a 3rd order model only. With only approximately 1,000 transitions per sequence a reliable estimation of such higher order processes would not have been possible. In this sense, there is the potential for our measure of complexity to not offer an accurate reflection of the absolute behavioural complexity. However, since this is a systematic bias, the relative differences in complexity between the different behavioural sequences, for example, collected in different circumstances should still reflect relative differences. For our purpose, differences in complexity between the behavioural sequences in different situations are more important than the actual level of complexity. Furthermore, we aimed at developing a measure of complexity that has the potential to be applied in practice and therefore needed to be practicable. We therefore did not consider increasing the duration of our behavioural sequences to longer than 24 h to obtain a greater number of changes for each transition. Additionally, a constant internal and external state of the animal and the environment would need to be presumed to produce valid results when increasing the observation period. The longer the observation period the less the likelihood of this being achieved.

The next step towards developing use of behavioural complexity as a welfare measure will be a practical validation of the approach in a specific data-set and testing the potential for automated measurement of behavioural complexity in the same data-set. We will therefore analyse real-life behavioural sequences of fattening pigs at different ages and in housing systems differing in their intensity. Generally, we advise including all behaviours in order to diversify the ethogram thereby increasing the chances of detecting differences in behavioural complexity. It might be tempting to exclude behaviours that are potentially linked to negative animal welfare (e.g. stereotypic or agonistic behaviour) since behavioural complexity is often viewed as an indicator for positive welfare given that it may reflect the behavioural capacity to deal with a specific situation. However, all behaviours are part of a continuous behavioural sequence upon which certain aspects of our approach to behavioural complexity is based. Moreover, it is likely that behavioural complexity decreases when an animal, for example, performs stereotypic behaviour as the number of behaviours, the number of transitions and the proportion of short behavioural states will decrease when an animal performs the same behaviour over an extended period of time. To a certain extent, agonistic behaviour is part of the natural behavioural repertoire and important to establish social hierarchies (Stolba & Wood-Gush 1989). Consequently, there is no basic and *a priori* reason to exclude such a behaviour from calculating behavioural complexity. An analogy to the previous argument would be that we may expect behavioural complexity to be reduced if agonistic behaviour becomes a major aspect in an animal’s behavioural sequence. In addition, there is no doubt that persistent agonistic behaviour in a group of pigs has negative welfare implications and requires action (O’Malley *et al.* 2022). We will therefore also include the mentioned behaviours in our ethogram for the next validation step. We will choose 11 different behaviours with the aim of achieving a balance between level of detail and feasibility. We will apply the 14 features of behavioural complexity presented in this study on the behavioural sequences of the fattening pigs. For the validation of the approach, we expect to find similar patterns of the loadings in the PCA indicating a consistent way of aggregation of the single measures. For the complexity measure itself we expect to see higher values for individuals in environments approaching more natural conditions compared to individuals in conventional and more intensive housing systems. For the automated measurement we will collect additionally continuous data on activity and movement patterns from 3-D accelerometers. We aim at extracting the features of behavioural complexity from the recorded activity patterns for a practical and automated application on-farm.

### Animal welfare implications

With further validation, behavioural complexity as an individual-based animal welfare indicator could extend beyond the currently used animal-based welfare indicators.There are indications that it does not only indicate states of pain and distress, for example, but also an animal’s capability of meeting its needs and motivations (Asher *et al.* 2009; Macintosh *et al.* 2011). Another advantage of behavioural complexity as an animal welfare indicator is its presumed sensitivity. A reduction in complexity may occur much earlier than a change in the duration or frequency of a behaviour as currently used in conventional animal-based indicators (Rutherford *et al.* 2006). Possibly, complexity can even be reflected by a relatively simple iceberg indicator, the Shannon diversity index of the frequency of different behaviours. In addition, behavioural complexity is an individual-based indicator and it is therefore also conceivable that it could function as an on-farm, early warning system on the state of single animals. The use of complexity as an individual-based welfare indicator could be further increased if it proves to be automatically detectable.

## Conclusion

We were able to devise an approach with the potential to construct a compact and aggregated measure of determining behavioural complexity. This measure reflected variations in complexity of artificially created behavioural sequences. The number of behavioural categories and the proportion of short transitions had the greatest influence on behavioural complexity. Our approach shows promise as a basis for further studies on behavioural complexity and may shed light on how complexity is related to (reduced) welfare.

## Supporting information

Raudies and Gygax supplementary material Raudies and Gygax supplementary material

## Data Availability

The study’s generated datasets and corresponding analysis code can be obtained under https://doi.org/10.17605/osf.io/g5rvu.

## Supplementary material

The supplementary material for this article can be found at http://doi.org/10.1017/awf.2024.48.
