# Supplementary material for: The construction of a measure of behavioural complexity as a potential individual-based welfare indicator and its theoretical validation
Source: Anim Welf. 2024 Nov 11;33:e51. doi: 10.1017/awf.2024.48 (PMC11655276; doi:10.1017/awf.2024.48)
Supplement: Raudies and Gygax supplementary material [file S0962728624000484sup001.pdf]

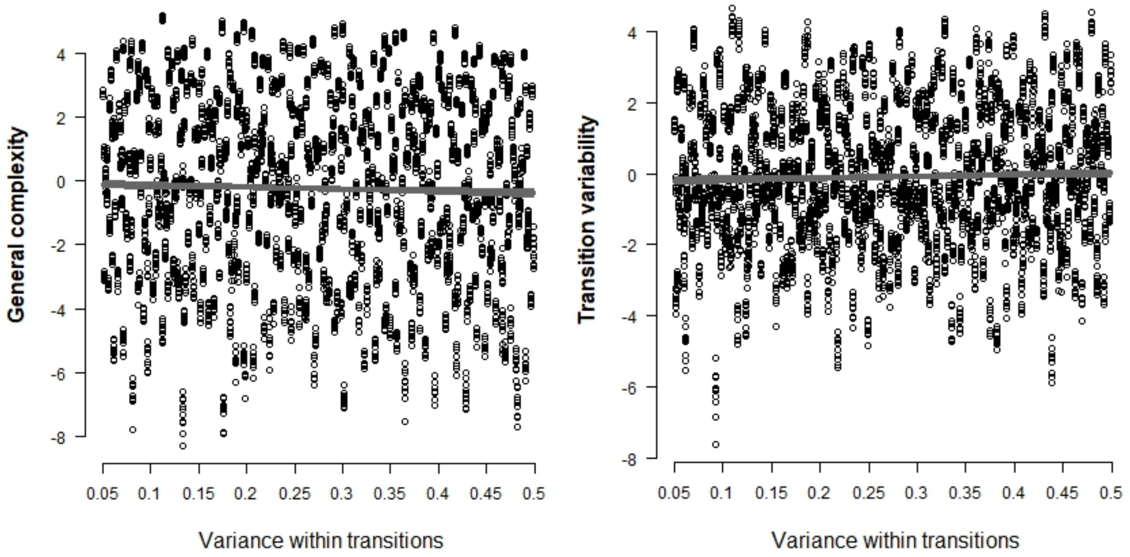

Figure S1. Effect of the relative variation among the short and long transitions on the general complexity (left) and the transition variability (right). The grey line shows the model estimation and dashed lines show the (very narrow) 95% confidence intervals.
